# The effects of citicoline on the level of consciousness, trend and plasma levels of malondialdehyde (MDA) and lipid profile in patients with head trauma suffering from “DAI (Diffuse Axonal Injury) with GCS≤8”

**Published:** 2012-11

**Authors:** Firooz Salehpoor, Ghaffar Shokouhi, Aydin Kazempoor Azar, Ali Baradaran Bagheri, Atta Mahdkhah, Farhad Mirzaei

**Affiliations:** ^*a*^Department of Neurosurgery, Tabriz University of Medical Sciences, Tabriz, Iran.

## Abstract

**Background:**

The present study aims to examine the pharmacotherapy effects of citicoline in patients suffering traumatic brain injury with DAI and GCS less than or equal to 8 diagnosis. The treatment efficacy of citicoline was assessed by the malondialdehyde (MDA) levels in plasma as a marker of oxidative stress and GCS clinical assessment of patients. Furthermore, because of the relation between MDA and lipid peroxidation, the lipid profile of patients was examined.

**Methods:**

After obtaining a medical TESTIMONIAL, the patients divided randomizely into 2 groups (case and control). Peripheral venous blood samples (10cc) were obtained from all patients in the first, sixth and twelfth days after admission to assess the levels of lipid profile and malondialdehyde. After obtaining the first sample, the patient of case group received injections of citicoline (500mg q 6h) for 15 days. During this period, the GCS score of the patients was determined and recorded by one researcher.

**Results:**

The MDA levels in different times of blood sampling were significantly different (P equal to 0.05), whereas control group showed no difference. In evaluation of lipid profile there was no significant difference at different times of blood sampling. The observed difference in the average plasma level of cholesterol had statistical significance between the two groups (P equal to 0.001), but no significant difference was found in other parameters of lipid profile (TG, HDL) as well as MDA. The GCS scores were significantly different at different times of post-admission (P equal to 0.001) in which the 15th day showed highest score, while, there was no statistical significant difference between GCS of two groups.

**Conclusions:**

The results of this study suggest that citicoline is an effective neuroprotective agent and can reduce MDA levels.

**Keywords:**

Citicoline, Diffuse Axonal Injury, Glasgow coma scale, Malondialdehyde, lipid profile


					Volume 4, Suppl. 1 Nov 2012
Publisher: Kermanshah University of Medical Sciences
URL: http://www.jivresearch.org
Copyright:
Congress of Iranian Neurosurgeons, 14-16 November 2012, Kermanshah, Iran
Paper No. 71
The effects of citicoline on the level of consciousness, trend
and plasma levels of malondialdehyde (MDA) and lipid
profile in patients with head trauma suffering from "DAI
(Diffuse Axonal Injury) with GCS8"
Firooz Salehpoor a
, Ghaffar Shokouhi a
, Aydin Kazempoor Azar a
, Ali Baradaran Bagheri a
,
Atta Mahdkhah a,*
, Farhad Mirzaei a
a
Department of Neurosurgery, Tabriz University of Medical Sciences, Tabriz, Iran.
Abstract:
Background: The present study aims to examine the pharmacotherapy effects of citicoline in patients suffering
traumatic brain injury with DAI and GCS less than or equal to 8 diagnosis. The treatment efficacy of citicoline was
assessed by the malondialdehyde (MDA) levels in plasma as a marker of oxidative stress and GCS clinical
assessment of patients. Furthermore, because of the relation between MDA and lipid peroxidation, the lipid profile
of patients was examined.
Methods: After obtaining a medical TESTIMONIAL, the patients divided randomizely into 2 groups (case and
control). Peripheral venous blood samples (10cc) were obtained from all patients in the first, sixth and twelfth days
after admission to assess the levels of lipid profile and malondialdehyde. After obtaining the first sample, the patient
of case group received injections of citicoline (500mg q 6h) for 15 days. During this period, the GCS score of the
patients was determined and recorded by one researcher.
Results: The MDA levels in different times of blood sampling were significantly different (P equal to 0.05), whereas
control group showed no difference. In evaluation of lipid profile there was no significant difference at different
times of blood sampling. The observed difference in the average plasma level of cholesterol had statistical
significance between the two groups (P equal to 0.001), but no significant difference was found in other parameters
of lipid profile (TG, HDL) as well as MDA. The GCS scores were significantly different at different times of post-
admission (P equal to 0.001) in which the 15th day showed highest score, while, there was no statistical significant
difference between GCS of two groups.
Conclusions: The results of this study suggest that citicoline is an effective neuroprotective agent and can reduce
MDA levels.
Key words:
Citicoline, Diffuse Axonal Injury, Glasgow coma scale, Malondialdehyde, lipid profile
* Corresponding Author at:
Atta Mahdkhah: Department of Neurosurgery, Tabriz University of Medical Sciences, Tabriz, Iran, Tel: 09144039122,
Email: mahdkhah@yahoo.com, (Mahdkhah A.).

				

